# Supplementary material for: Acceptance and compliance with micronutrient powder (MNP) among children aged 6–23 months in northern Nigeria
Source: PLOS Glob Public Health. 2022 Oct 17;2(10):e0000961. doi: 10.1371/journal.pgph.0000961 (PMC10022258; doi:10.1371/journal.pgph.0000961)
Supplement: S1 Dataset — (ZIP) [file pgph.0000961.s008.zip › S8_Dataset/In-depth Interviews.zip!/In-depth Interviews/030715_A_SO_ID_2_CG.pdf]

0107115_A_SO _ID_CG_2.doc

In depth interview with caregiver in Bango gari community of Song Local Government Adamawa State By Ishaku David Saraso on the 1st July 2015

Duration _53m 56S.

Introduction

1_ Describe for me the people in your immediate family living in your household

R_ We are living with my husband and six children and my husband elder brother with his wife and two children my husband junior sister with her four children.

I_ Tell me about specific gender and age of the children 6 – 23 Months

R_ It is only my child who is 15 Months and he is a Male child.

CHILDREN 6 – 23 MONTHS

I _ We have heard of food challenges for young children in this setting related to the seasons.

R_ At the time of harvest we have enough of ground nut to prepare pap for the Child but now that it is planting time the price has rise so we don't have enough to prepare pap.

I _ how do you cope with this food challenges, related to seasons.

R_ I use to buy ground nut for N20 or N50 to make sure I prepare the Pap to give my Child MNP.

I_ Where do you get the money to buy ground nut?

R_ I use to sell food in the morning and evening and I have provision shop.

I_ can't you tell me how challenges affect infant and young Child feeding

R_ As I said before it affect infant and young Child feeding because when the price of groundnut is low that is harvest time we use to prepare Pap with eight groundnut with a little flower but now that the price is high we use very little groundnut to prepare Pap for the Child .

I _ One thing we heard in other interview that caregivers often give their Children 6 – 23 months 'tea' could you defined explain tea?

R_ Tea is made up of hot water, Lipton sugar and milk (cowbell)

EXPERIENCE WITH MNP DURING TRIAL (STORIE)

I _ Now I want us to talk about the MNP that you receive. Could you tell me about your experience with the produce?

R_ I have seen many things because before I start given him MNP the child don't like eating food the sucking breast the only thing he like is water but now that we are given him MNP he eat food suck breast whatever he see if it is food he like to eat.

POSITIVE ASPECTS OF USING MNP

I_ Please can you talk about any positive aspects of the experience with MNP.

R_ There are many changers in the child his weight has increase because many people that use to carry him always ask me what I am given my child that make him add weigh (more than 5 people) so there is difference in his weight now and before using the MNP secondly MNP has help in his body building.

I_ Tell me perceived benefit of this MNP to the Child.

R_ Before using MNP the child don't use to be eaten food or suck breast and he use to be ill most of the time, but now that we are using it he eat food, such breast and he don't fall sick any how like before.

I_ Tell I more about specific stories related to improvement in Child health compared to before using the MNP

R_ Now that we are using MNP the child dance, play with his brother and whatever he want to do he use to do it with force like some days back I cook food and keep for his father before the farther wash his hand the child pour the food on the ground.

CHALLENGES/BARRIERS USING MNP

I_ Talk about any general challenges you had during the MNP trial.

R_ When the community health worker gave us the MNP the first and the second day that we start using it his body was hot and he fells week, secondly the pap that is mix with MNP he don't like to be take it unless I lay him on the bed and feed him, but the one that is not mix with MNP he will collect the cup of Pap and take it, I don't know either he has notice something.

I_ when you mixed the MNP with Pap do the child see it

R_ Yes because I use to mixed it with the child on my leap

I_ You said the first two day of using MNP the child body was hot and week how did you deal with the effects.

R_ I gave the child paracetamol and when I notices it was MNP I stop given him the paracetamol.

I_ some caregivers mentioned that it was hard to store the MNP without it being taken by others or eaten by rodents. Did you have any similar experience?

R_ No because I store the MNP is our cultural pot which is use for decoration it is cover with calabash and when I want to use it I make sure that his brothers and sisters did not see me taken it out from the pot.

I_ Some mothers indicated that it might be hard to remember to use MNP. Did you have a similar experience and what did you do to remember.

R_ No because in the evening I make sure that I have Pap in the flaks that I will mixed MNP in the morning to give my child.

I_ Can you tell me specific solutions that you think or suggest to address above mentioned barrier or challenge

R_ I don't think there is any barrier or challenge because the way MNP has improved my child health it make me not to see it as barrier or challenge, and I have made up my mind that I will give my child the MNP.

CHILD FEEDING BEHAVIOURS AND RESPONSE TO MNP

I_ Let's talks about the child feeding and response to the MNP. Talk about how you feed the MNP to the child usually.

R_ In the morning when he weak up I was his face and brush his teeth and put him on the purr to pass stool then I was hi anus. I will get Pap in a cup and make it to be cool and I will get like two to three spoons and mix with the pap and I will lay him on the bed and feed him with the pap mix with MNP when I finish feeding him with that I will then give him the pap that is not mix with MNP he will take it himself in a cup.

I_ Please tell me when the MNP was usually given to the child

R_ I use to give him MNP in the morning and ones a day

I_ can't you tell me either your Child like or dislikes the taste of MNP

R_ At the beginning when he see it he will be crying but I use to lay him on the head to feed him with the, mix MNP. But Pap that is not mix with MNP he will collect it in a cup and take it

I_ Please tells me how MNP consumption impacted the child's current diet.

R_ MNP has increased my child appetites which make him to take breast milk, and consumption varieties of food.

SHARING MNP

I_ In other communities we noticed that sharing of the MNP occurred. Could you talk about any sharing of the MNP when you used it?

R_ when the community health workers gave us MNP they tell us that when MNP is given to use it not expected to be shared with any other person because they ask us about the age (month) of the child and they also warm us no sharing with another person or his child or the Childs brother or sister. Secondly we should keep (store) MNP where their elder brothers and sisters will not see it or take so we follower the instruction of the community health workers.

I_ Tell me about MNP sharing due to food shortage during lean season

R_ we are not fasten so there is not thing like food sharing during lean season.

I_ Please tell me whether caregiver or father ate the MNP as a snack

R_ we have not eat MNP since the community health workers instructed us not to eat, but CMAN some caregiver use to eat it as their food and drink water.

I_ Tell me whether left over of the MNP being consumed by others.

R_ No because I use to get three (3) to five (5) spoons and mix with MNP and then give the child so there was no left over of MNP.

I_ will 60 sachets of MNP be enough for 60 days why/why not

R_ Yes because I just use 15 sachets of MNP in 15 days and one per day for 60 days.

PERCEPTIONSOF THE MNP

I_ Now that you have used MNP, could you talk about how you perceive it?

R_ The MNP is good especially to me because my child don't have appetite of eaten anything, we have used so many drugs like vitamin C and Multivite to make him eat food since the time that he started eaten food (6month) but there is not any change, that is why I keep on say that the government should not stop given MNP. They should continue to give to those that have not gotten the MNP because it is good for the health of our children.

I_ Please tells me whether you see MNP more as a food, a medicine or something else why?

R_ I sees it as medicine because it is just as the way a growth up person is ill and lost appetite he will look for motivate to make him eat food, that is why I see MNP a medicine.

WILLINGNESS TO PAY FOR MNP

I_ Please will you purchase this product (MNP) if cheaply on sale in your community.

R_ Yes because it has improved my child health condition

I_ Tell me more

R_ The weight of the child had increased and he don't use to be ill like before.

I_ How much would you be willing to pay to each sachet?

R_ Since I have tested and know its important I can even pay a sachet at the cost of ₦50

SOCIAL MARKETING THE MNP

I_ You are giving me great information now please tell me some ways that you believe would be effective for promoting MNP to caregivers in the community so that they would comply with using it appropriately?

R_ My advice is that they should fixed a day for sharing MNP like that of CMAN and IYCS so that the commonly health workers will make sure the child is at the age (month) of 6 – 23 before they will give to the caregiver.

I_ Tell me way that would be effective to promote MNP for its appropriate use.

R_ When the government brings this product MNP they should send message around to community that there is a product that is good for their children and the product will share in a specific place at given time and day I believe the caregivers will come out for that.

I_ Who influences your decisions regarding feeding your baby the MNP?

R_ It was one of the community health workers because she is my classmate in my secondary school.

I_ Tell me good place where health information related to MNP would be disseminate.

R_ I think the best place where health information related to MNP would be disseminated is the community health center (office) in the mosque, church and throw town crier.

I_ Way

R_ Because we trust our community health worker and religious leaders because they will not bring what will harm our children's.

I_ Who in your community do you trust the most for receiving messages about infant and young child feeding.

R_ Is the community health workers because we believe and have confident in them.

I_ What form of media do you prefer of receiving MNP Message?

R_ Radio

I_ Tell me ways you preferred of received education regarding IYCF and MNP

R_ Community health workers

I_ Why community health workers

R_ Because the product MNP is related to health and most of the time when something come up like this it is the community health workers that health educate us.

I_ Tell me more about successful health education campaign in this community?

R_ Ok like this CMAN the community health workers, volunteer, community head/ ward heads and religious leaders where all involves and it was successful.

I_ What made it successful?

R_ Because it involves community health workers, community/ward head and religious leaders.

I_ Tell me the best community channels to distribute the product.

R_ The best channel is through the community health workers

I_ Why

R_ Because they have been given use health education and talks and also given us product in the pass which has so much improved our health and our children health, so we believe in them.

I_ I want to hear some specific massages ages you think would effectively promote the product

R_ You should tell the caregivers that MNP is very good for their children, it will improve their child health increase the weight of the child because it will give them appetite to eat and also protect their child from illness it has no side effect.

I_ Please tell me effective messages to promote its use but limit the sharing of it

R_ In state of given the MNP two packets let them be given one packet when the one finish the they give the other one.

FINAL THOUGHTS

I_ Finally is there anything else that you can share about your experience with this product?

R_ As I have said before if it were cheaply on sale in her community I can even purchase more than ₦ 50 I have seen it benefit to my child so it is a welcome idea.
